# RhoA Drives T-Cell Activation and Encephalitogenic Potential in an Animal Model of Multiple Sclerosis

**DOI:** 10.3389/fimmu.2018.01235

**Published:** 2018-05-31

**Authors:** Alba Manresa-Arraut, Flemming Fryd Johansen, Cord Brakebusch, Shohreh Issazadeh-Navikas, Henrik Hasseldam

**Affiliations:** ^1^Neuroinflammation Unit, Biotech Research and Innovation Centre (BRIC), Faculty of Health and Medical Sciences, University of Copenhagen, Copenhagen, Denmark; ^2^Cytoskeletal Organization Group, Biotech Research and Innovation Centre (BRIC), Faculty of Health and Medical Sciences, University of Copenhagen, Copenhagen, Denmark

**Keywords:** RhoA, multiple sclerosis, experimental autoimmune encephalomyelitis, T-cell, neuroinflammation

## Abstract

T-cells are known to be intimately involved in the pathogenesis of multiple sclerosis (MS) and its animal model experimental autoimmune encephalomyelitis (EAE). T-cell activation is controlled by a range of intracellular signaling pathways regulating cellular responses such as proliferation, cytokine production, integrin expression, and migration. These processes are crucial for the T-cells’ ability to mediate inflammatory processes in autoimmune diseases such as MS. RhoA is a ubiquitously expressed small GTPase well described as a regulator of the actin cytoskeleton. It is essential for embryonic development and together with other Rho GTPases controls various cellular processes such as cell development, shaping, proliferation, and locomotion. However, the specific contribution of RhoA to these processes in T-cells in general, and in autoreactive T-cells in particular, has not been fully characterized. Using mice with a T-cell specific deletion of the RhoA gene (RhoA^fl/fl^LckCre^+^), we investigated the role of RhoA in T-cell development, functionality, and encephalitogenic potential in EAE. We show that lack of RhoA specifically in T-cells results in reduced numbers of mature T-cells in thymus and spleen but normal counts in peripheral blood. EAE induction in RhoA^fl/fl^LckCre^+^ mice results in significantly reduced disease incidence and severity, which coincides with a reduced CNS T-cell infiltration. Besides presenting reduced migratory capacity, both naïve and autoreactive effector T-cells from RhoA^fl/fl^LckCre^+^ mice show decreased viability, proliferative capacity, and an activation profile associated with reduced production of Th1 pro-inflammatory cytokines. Our study demonstrates that RhoA is a central regulator of several archetypical T-cell responses, and furthermore points toward RhoA as a new potential therapeutic target in diseases such as MS, where T-cell activity plays a central role.

## Introduction

Multiple sclerosis (MS) is a chronic immune-mediated disorder of the central nervous system (CNS) that leads to demyelination and neurodegeneration of gray and white matter (1–3). It is widely established that T-cells play an important role in the pathogenesis of MS (2, 4, 5). Both MS and its animal model, experimental autoimmune encephalomyelitis (EAE), are suggested to be mediated by Th1 and Th17 cells and share essential pathological and clinical features with MS such as multifocal lymphocytic infiltrations, CNS inflammation, demyelination, and axon degeneration, which lead to paralysis and sensory disturbances (1, 6).

Therapies targeting immune cells have proven effective in MS patients. Antibody therapy with Natalizumab is the second-line treatment for MS after interferon-β (IFN-β) and Glatiramer acetate (7, 8). It targets the α4β1 integrin of lymphocytes, thereby inhibiting their migration across the blood–brain barrier (BBB) (9–11). Depletion of lymphocytes after treatment with the anti-CD52 antibody Alemtuzumab dramatically reduced lymphocyte numbers in peripheral blood in RRMS patients, thereby stopping the inflammatory and autoimmune process from causing further CNS damage, resulting in decreased relapse rates (12, 13). This indicates that indeed the prevention of immune cell infiltration is a promising therapeutic strategy for neuroinflammatory diseases such as MS.

T-cell infiltration into the CNS is a tightly regulated multistep process. T-cells migrate from the blood circulation through the CNS barriers into the CNS, where T-cells secrete chemokines and pro-inflammatory cytokines that trigger BBB breakdown and facilitate further T-cell infiltration (3).

Cell migration is dependent on cytoskeletal rearrangements, which are in turn regulated by small Rho GTPases (14). To the best of our knowledge, the role of T-cell-specific RhoA has not been addressed in the context of EAE. More specifically, no T-cell conditional knock-out mice for RhoA have been studied to investigate if RhoA is essential for transmigration of pathogenic T cells *via* BBB to induce neuroinflammation in models of MS. However, several studies have been conducted using various *in vitro* systems. For example, a siRNA screen of all Rho GTPases identified RhoA as the main player in T-cell transendothelial migration *in vitro* (15), and experiments using the exoenzyme C3 transferase, which inhibits the GTPases RhoA, RhoB, and RhoC, also demonstrated the importance of these Rho GTPases in monocyte transendothelial migration *in vitro* (16). It has also been shown that inhibition of Rho signaling with C3 transferase results in reduced thymic cellularity, reduced numbers of mature single positive (SP) T-cells in the periphery and impaired T-cell clonal expansion in mice (17–19). These observations are tightly linked to the important role of Rho GTPases in cell migration, as T-cell maturation is regulated as they migrates through the thymus (20), and this migration in turn has been shown to be dependent on RhoA and its downstream effector ROCK (21, 22). Of note, inhibition of ROCK has shown beneficial effects in EAE (23), underscoring the therapeutic potential of RhoA for MS treatment and stressing the need for further research on the biological role of RhoA in T-cells during neuroinflammation.

These studies indicate that RhoA is important for T-cell activation and migration. However, the direct impact of RhoA on T-cell activation and transmigration from blood into CNS, resulting in CNS inflammation, has not been explored *in vivo*. In this study, we investigated whether specifically knocking out the small GTPase RhoA in T-cells had an impact on T-cell activation and functionality in EAE mice. We show that the lack of RhoA expression in T-cells results in a reduction in EAE incidence and a significant amelioration of disease severity, which coincides with a reduced RhoA^−/−^ T-cell infiltration into the CNS. Further characterization suggests that this could be due to decreased activation and attenuated encephalitogenic profile of RhoA^−/−^ T-cells, shown by a decreased T-cell proliferation and cytokine production. Furthermore, RhoA^−/−^ T-cells present a significantly impaired transendothelial migratory capacity. Taken together, our results suggest that RhoA is central for T cell proliferation and activation as well as their trans-BBB migratory capacity, and therefore could serve as a potential therapeutic target for neuroinflammatory diseases such as MS.

## Materials and Methods

### Experimental Animals

To generate mice with a T-cell-restricted deletion of the RhoA gene (RhoA^fl/fl^LckCre^+^), RhoA^fl/fl^ (24) and LckCre^+v^ (25) mice were crossed. Mice were kept as 129Sv/C57Bl/6 outbred strains. RhoA^fl/fl^LckCre^−^, RhoA^fl/+^LckCre^+^, and RhoA^fl/fl^LckCre^+^ mice were bred and housed in the AAALAC certified animal facility at the Biotech Research and Innovation Center, University of Copenhagen. Mice were housed two to six animals per IVC cage with free access to food and water on a 12-h light/dark cycle. All procedures were approved and performed in accordance with the Danish Animal Experimentation Committee (#2012-DY-2934-00001) and the European Council Directive #86/609 for the Care of Laboratory Animals.

### Induction and Evaluation of EAE

Experimental autoimmune encephalomyelitis was induced in 9- to 16-week-old female mice (RhoA^fl/fl^LckCre^−^ *n* = 39, RhoA^fl/+^LckCre^+^ *n* = 14, and RhoA^fl/fl^LckCre^+^ *n* = 12) by active immunization with MOG_35–55_ following the manufacturer’s protocol (Hooke labs, Lawrence, KS, USA). Age-matched mice were used in all experiments. Briefly, mice were injected subcutaneously (s.c.) in the upper and lower back with 200 µg of MOG_35–55_ emulsified in complete Freund’s adjuvant, followed by the intraperitoneal administration of 100 µl of 4 µg/ml pertussis toxin at 2 and 24 h post-immunization. Mice were monitored daily for clinical signs of disease and assigned a disease score according to the EAE clinical scoring system (0: no clinical symptoms to 5: moribund or dead) devised by the Danish Animal Experiments Inspectorate. When animals reached a score 3, food and water-gel was provided at the bottom of the cage. If animals reached a score 4 or above, or if weight loss exceeded 20%, the animals were euthanized. To allow statistical evaluation of all animals, accounting also for those that never developed disease throughout the experimental period, the day of disease debut of mice that never developed EAE was considered as 1 day after the sacrifice date, i.e., days 42, 43, and 50 post-immunization. Relapses were defined as an increase of the clinical score after the acute phase of disease by at least one point on the scoring scale (remission phase), followed by a worsening of disease of a minimum of one point on the scoring scale, as described previously (26).

### Tissue Preparation

Mice were sacrificed at different stages of the EAE disease course. At the end of the experiment, EAE mice were anesthetized with 5% isoflurane (Forene, Abbot Scandinavia, Stockholm, Sweden) delivered in pure oxygen and perfused transcardially with phosphate-buffered saline (PBS) until blood was cleared from circulation. Brains were collected and snap-frozen in isopentane, kept on dry ice, and embedded in Tissue-Tek^®^ (Sakura Finetek Denmark ApS, Vaerløse, Denmark). Using a cryostat, 3-µm sagittal sections of tissue were collected from four different levels with 400-µm distance in between. Sections were mounted on superfrost plus slides (Thermo Fisher Scientific, MA, USA) for histological or immunohistochemical staining.

For the *in vitro* functional assays of naïve T-cells, splenic CD3^+^ T-cells were isolated using a MACS Pan T-cell isolation kit II mouse (Miltenyi Biotec, Bergisch Gladbach, Germany). T-cells were cultured in complete culture medium composed by RPMI 1640 (Thermo Fisher Scientific), 10% FCS supplemented with IL-2 (5 ng/ml), and stimulated with plate-bound anti-CD3 and soluble anti-CD28 at the concentration and time specified in each assay.

For the *in vitro* functional assays of effector/memory T-cells, RhoA^fl/fl^LckCre^−^ and RhoA^fl/fl^LckCre^+^ mice were immunized with MOG_35–55_ following the manufacturer’s protocol (Hooke labs, Lawrence, KS, USA) as described above. 11 days after immunization, mice were anesthetized with 5% isoflurane (Forene) delivered in pure oxygen and perfused transcardially with PBS until blood was cleared from circulation. Spleens were collected and single-cell suspensions were prepared by mechanical disruption through 40-µm cell strainers. Splenocytes were cultured in complete culture media composed by RPMI 1640 (Thermo Fisher Scientific), 10% FCS supplemented with IL-2 (5 ng/ml), and 0.01% 2-Mercaptoetanol (Sigma-Aldrich) and stimulated with MOG_35–55_ (50 µg/ml) for 24–72 h, depending on the assay.

### Immunohistochemistry

Four brain sections covering the brain from the midline to the inner layers of the cortex were randomly chosen for CD3 staining. Briefly, sections were fixed in acetone at −18°C, rinsed in PBS containing 0.05% Tween-20 (PBS-T) (Gibco, Thermo Fisher Scientific), and subsequently blocked with PBS-T containing 5% goat serum (DAKO, Glostrup, Denmark) for 30 min at room temperature (RT). The sections were incubated for 1 h at RT with monoclonal rabbit anti-mouse CD3 antibody (1:100, Abcam clone SP7, Cambridge, UK). The sections were rinsed in PBS-T and incubated for 1 h at RT with horseradish peroxidase-conjugated polyclonal goat anti-rabbit IgG secondary antibody (1:400, Abcam). Then sections were rinsed with PBS-T and incubated with diaminobenzidine for 30 s. Sections were rinsed again in PBS-T before counterstaining with hematoxylin for 20 s and dehydration through increasing concentrations of ethanol (70, 96, and 99%) and xylene. Sections were mounted with DPX mounting media (Cellpath, Newtown, UK).

### T-Cell Quantification

The stained sections were scanned using a NanoZoomer-2.0HT (Hamamatsu, Japan) at 40× magnification, and the micrographs were used for quantification of CD3^+^ cells. The cells were manually counted in the following areas: cerebral parenchyma, meninges, ventricular system, and perivascular space (PVS). The anatomical regions were identified using the hematoxylin staining. All sections were quantified by two blinded observers using the QuPath open source software for digital pathology image analysis (https://qupath.github.io/) (27).

### Flow Cytometric Analysis of Spinal Cords of EAE Mice

Mice at the chronic phase of the EAE disease (36–56 days post-immunization) were sacrificed, spinal cords were collected, and single-cell suspensions were prepared by mechanical disruption through 40-µm cell strainers. Single-cell suspensions were stored in DMEM medium (Thermo Fisher Scientific) supplemented with 10% FCS and 10% DMSO at −80°C until used. Cell suspensions were quickly thawed at 37°C, myelin was removed using a MACS Myelin Removal Beads II (Miltenyi Biotec), and cells were stained for flow cytometry. Briefly, 5 × 10^5^ cells in 50 µl of cell staining buffer (BioLegend, San Diego, CA, USA) were incubated with the viability marker Zombie UV™ (BioLegend) for 20 min at RT. Cells were subsequently washed, resuspended in 50 µl of cell staining buffer, stained with American hamster anti-mouse TCR AF700 (1 μl/test, BioLegend clone H57-597) and rat anti-mouse CD4-PE-Cy5 (0.3 µl/ml, eBioscience, clone GK1.5) or isotype controls for 30 min on ice. Cells were then washed, resuspended in fixation/permeabilization working solution (eBioscience), and incubated for 30 min at RT. After washing, cells were resuspended in 50 µl cell staining buffer and stained with rat anti-mouse IL-4 PE (2 μl/test, BD Biosciences, clone 11B11), rat anti-mouse IL-17A FITC (1 μl/test, eBioscience clone eBio17B7), rat anti-mouse TNFα APC (2 μl/test, BD Biosciences clone MP6-XT22), rat anti-mouse IFNγ PE (2 μl/test, BD Biosciences clone XMG1.2), rat anti-mouse GM-CSF FITC (5 μl/test, eBioscience clone MP1-22E9), and rat anti-mouse CD11b FITC (1 μl/test, BD Biosciences clone M1/70) for 30 min on ice. Subsequently, cells were washed, resuspended in 400 µl of cell staining buffer, and analyzed by flow cytometry. Flow cytometry was performed using the LSRII (BD Biosciences) and the CellQuest software (BD Biosciences). Data analysis was done using the FlowJo V10 software (Tree Star). Isotype controls were included as negative controls and subtracted.

### Proliferation Assay

Spleens were isolated from naïve RhoA^fl/fl^LckCre^−^ and RhoA^fl/fl^LckCre^+^ mice, and single-cell suspensions were prepared by mechanical disruption through 40-µm cell strainers. Naïve CD3^+^ cells were enriched by magnetic cell sorting as described above and labeled with Green Cell Trace™ CFSE (Thermo Fisher Scientific), 0.5 µM for 10^7^ cells. Labeled cells were cultured in 96-well plates (10^6^ cells/ml, Corning Costar, Tewksbury, MA, USA) and stimulated with plate-bound anti-mouse CD3 (5 µg/ml, BioLegend clone 145-2C11) and soluble anti-mouse CD28 (2 µg/ml, BioLegend clone 37.51) for 72 h in culture. Subsequently, cells were stained with the viability marker 7-AAD (BioLegend) and analyzed by flow cytometry with the FACS Calibur (BD Bioscience). Data analysis was done using the FlowJo V10 software (Tree Star).

For the analysis of proliferation of effector/memory T-cells, spleens were isolated from RhoA^fl/fl^LckCre^−^ and RhoA^fl/fl^LckCre^+^ mice immunized with MOG_35–55_ 11 days earlier, and single-cell suspensions were prepared as described above. CFSE-labeled splenocytes were re-stimulated with MOG_35–55_ (50 µg/ml, Sigma-Aldrich) for 48 h in culture, and proliferation was analyzed as described above.

### Cell Viability Analysis

The Vybrant™ FAM Caspase-3 and -7 Assay Kit (Life Technologies, Thermo Fisher Scientific) was used to evaluate cell viability by simultaneously analyzing caspase activity and cell membrane permeability by flow cytometry. Naïve CD3^+^ enriched T-cells were isolated as described above and stimulated for 24 h with plate-bound anti-mouse CD3 (3 µg/ml, BioLegend) and soluble anti-mouse CD28 (3 µg/ml, BioLegend). Effector/memory T-cells were isolated from previously MOG_35–55_-immunized mice as described above and stimulated with MOG_35–55_ (50 µg/ml, Sigma-Aldrich). Cells were then stained following the manufacturer’s protocol. Briefly, 300 µl of cell suspension at a concentration of 10^6^ cells/ml were incubated with FLICA 30× working solution during 1 h at 37°C in the dark. Subsequently, cells were washed and incubated on ice with 2 µl of PI for at least 5 min before flow cytometric analysis on the LSRII (BD Biosciences). Data analysis was done using the FlowJo V10 software (Tree Star).

### Determination of T-Cell Activation State

Briefly, 5 × 10^5^ cells in 50 µl of cell staining buffer (BioLegend) were incubated with rat anti-mouse CD4-PE (0.2 µg/test, BD Pharmingen clone GK1.5) and rat anti-mouse CD25-PE/Cy5 (0.125 µg/test, eBioscience clone PC61.5) or isotype controls for 30 min on ice. Subsequently, cells were washed, resuspended in 300 µl of cell staining buffer, stained with the viability marker 7-AAD (BioLegend), and analyzed by flow cytometry. Flow cytometry was performed using the FACS Calibur (BD Biosciences) and the CellQuest software (BD Biosciences). Data analysis was done using the FlowJo V10 software (Tree Star). Isotype controls were included as negative controls and subtracted.

### Determination of Cytokine Production *In Vitro*

Culture supernatant from RhoA^+/+^ and RhoA^−/−^ CD3^+^ T-cells was collected after 24, 48, and 72 h of stimulation with plate-bound anti-mouse CD3 (3 µg/ml, BioLegend) and soluble anti-mouse CD28 (3 µg/ml, BioLegend) in case of naïve T-cells, or with MOG_35–55_ (50 µg/ml, Sigma-Aldrich) for effector/memory T-cells isolated from mice immunized with MOG_35–55_ 11 days before. We used the Essential Th1/Th2 Cytokine 6-plex Mouse ProcartaPlex™ Panel (Invitrogen, Thermo Fisher Scientific), which targets the mouse cytokines IFN-γ, IL-12p70, IL-6, TNF-α, IL-4, and IL-5. For the determination of IL-17A, the Mouse IL-17A uncoated ELISA kit (Invitrogen) was used. Each sample was processed according to the manufacturer’s instructions. Samples were analyzed in duplicates, and cytokine concentrations were interpolated from the standard curve. The multiplex plate was read in the Bio-Plex^®^ MAGPIX™ Multiplex reader (BioRad, Hercules, CA, USA) and data acquired using the Bio-Plex Manager Software v6.1. The ELISA plate was read in the GloMax^®^-Multi Microplate Multimode Reader (Promega, Madison, WI, USA).

### Transmigration Assay

The T-cell transmigration assay was performed in polycarbonate Transwell™ filter inserts of 8-µm pore size (Corning Costar) covered with a confluent monolayer of mouse brain endothelial bEnd.3 cells. bEnd.3 cells (passage number > 30) from confluent 10 cm^2^ dishes (Corning Costar) were trypsinized and plated onto the Transwell™ filter inserts at a seeding density between 5 × 10^4^ and 10^5^ cells/cm^2^ in 100 µl of DMEM medium (Thermo Fisher Scientific) supplemented with 10% FCS, penicillin G (100 U/ml), and streptomycin (100 µg/ml). Culture medium was changed every 48–72 h. Splenocytes were CD3^+^ enriched by magnetic sorting as described above and activated with plate-bound anti-CD3 (3 µg/ml, BioLegend) and anti-CD28 (3 µg/ml, BioLegend) for 24 h. When bEnd.3 cells were confluent after 5 days of culture, the culture medium was removed and 100 µl of activated T-cells (5 × 10^4^ T-cells/well) in serum-free 1640 RPMI medium were placed in the upper chamber, and 600 µl of fresh serum-free 1640 RPMI medium was added in the bottom chamber of the Transwell™. The plates were then incubated at 37°C in 5% CO_2_ in the IncuCyte ZOOM^®^ (Essen BioScience, Ann Arbor, MI, USA) for live-cell time lapse imaging of the transmigrated cells. Nine images per well from three technical replicates were taken every 30 min for 4 h using a 20× objective lens and then analyzed using the IncuCyte™ Basic Software. In phase contrast, cell segmentation was achieved by applying a mask to exclude cells from background. An area filter was applied to exclude objects below 10 µm^2^. After 4 h, the transmigrated T-cells present in the bottom chamber were recovered and quantified using the Accuri™ C6 flow cytometer (BD Biosciences, San Jose, CA, USA). Data analysis was done using the FlowJo V10 software (Tree Star).

### Statistics

Quantitative data are presented as mean ± SEM. All analyses were performed using Prism 7 (GraphPad Prism software) and considered significant at *p* ≤ 0.05. Differences in the EAE disease incidence, disease debut, and EAE clinical score (including maximum clinical score and mean score during the acute phase) were analyzed using Kruskal–Wallis test with Dunn’s multiple comparisons test. One-way ANOVA with Dunnett’s multiple comparisons test was used to study the differences in the cumulative EAE clinical score. Unpaired Student’s *t*-test was used to evaluate the differences in CD3^+^ cells infiltrated in EAE brains. To analyze differences in the proliferation index, two-way ANOVA with Tukey’s multiple comparisons test was used. Multiple *t*-test corrected for false discovery rate (FDR) with the Benjamini, Krieger, and Yekutieli method was used to evaluate differences in cell viability (apoptosis and necrosis rates). Differential cytokine production analysis was done using two-way ANOVA with Sidak’s multiple comparisons test and unpaired Student’s *t*-test for two-group comparisons. The study of the T-cell activation capacity was done using one-way ANOVA with Tukey’s multiple comparisons test. Differences in the migratory capacity were analyzed using two-way ANOVA with Sidak’s multiple comparisons test and unpaired Student’s *t*-test for two-group comparisons.

## Results

To study the specific role of RhoA in T-cells, mice with a conditional knock-out of the RhoA gene were generated using the Cre-loxP system, as previously reported (28). To knock out RhoA specifically in T-cells, Cre recombinase was under the control of the lymphocyte-specific Lck promoter. We obtained a knock-out efficiency of ~85% (Figure S1 in Supplementary Material) and observations on weight, behavior, and vitality of healthy RhoA^fl/fl^LckCre^+^ mice did not differ from wild-type littermates (RhoA^fl/fl^LckCre^−^ mice).

Flow cytometric analysis of the T-cell pool of naïve RhoA^fl/fl^LckCre^−^, RhoA^fl/+^LckCre^+^, and RhoA^fl/fl^LckCre^+^ mice showed that lack of RhoA in T-cells had no impact on the numbers of blood circulating lymphocytes, CD3^+^ cells, SP CD4^+^ and CD8^+^ cells, double positive (DP) CD4^+^CD8^+^ cells, and double negative (DN) CD4^−^CD8^−^ cells (Figures S2B–G in Supplementary Material, respectively). In thymus of RhoA^fl/fl^LckCre^+^ mice, we observed reduced numbers of lymphocytes, CD3^+^ cells, SP cells, and DP cells (Figures S2H–L in Supplementary Material, respectively), and increased numbers of DN cells (Figure S2M in Supplementary Material) compared with RhoA^fl/+^LckCre^+^ and RhoA^fl/fl^LckCre^−^ mice, suggesting defects in early T-cell development. In spleen, lymphocyte populations did not differ between mice from different genotypes (Figure S2N in Supplementary Material), while CD3^+^ cells and SP cells (Figures S2O–Q in Supplementary Material, respectively) were found significantly reduced in RhoA^fl/fl^LckCre^+^ mice compared with RhoA^fl/+^LckCre^+^ and RhoA^fl/fl^LckCre^−^ mice, suggesting that other lymphocytes such as B-cells and NK cells compensated for the lack of T-cells in spleen.

These results suggest that RhoA is to some degree indispensable for T-cell development, even though the peripheral blood T-cell pool was unaffected. Thus, we wondered whether lack of RhoA also impacts archetypical T-cell responses such as proliferation, cytokine production, and migration, in an autoimmune setting.

### Lack of RhoA Expression in T-Cells Decreases EAE Disease Severity

**Figure 1:**
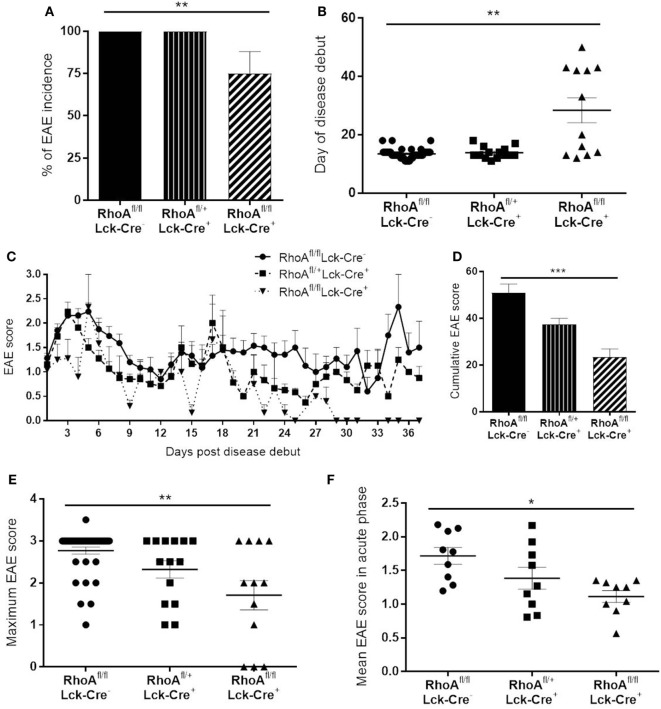
Lack of RhoA in T-cells reduces experimental autoimmune encephalomyelitis (EAE) disease incidence, onset and severity. **(A)** Disease incidence in RhoA^fl/fl^LckCre^−^, RhoA^fl/+^LckCre^+^, and RhoA^fl/fl^LckCre^+^ mice. **(B)** Day of disease debut of mice from the three different genotypes. **(C)** EAE disease profile after disease onset in RhoA^fl/fl^LckCre^−^, RhoA^fl/+^LckCre^+^, and RhoA^fl/fl^LckCre^+^ mice. **(D)** Overall EAE disease severity in the three different genotypes, represented as the cumulative EAE score over the entire study period. **(E)** Maximum disease score reached by each mouse throughout the experiment. **(F)** Mean disease score of each mouse during the acute phase of the disease (days 1–9 after disease debut). RhoA^fl/fl^LckCre^−^ *n* = 39, RhoA^fl/+^LckCre^+^ *n* = 13, RhoA^fl/fl^LckCre^+^ *n* = 12. Data shown as mean ± SEM. *p < 0.05, **p < 0.01 (Kruskal–Wallis test followed by Dunn’s test).

At first, we wanted to determine whether lack of RhoA expression in T-cells would affect disease initiation and/or severity of EAE. We induced chronic demyelinating EAE in 129Sv/C57Bl/6 mice by injection of emulsified myelin oligodendrocyte glycoprotein (MOG_35–55_). We observed a significantly decreased disease incidence in RhoA^fl/fl^LckCre^+^ mice compared with its heterozygote and homozygote wild-type littermates, RhoA^fl/−^LckCre^+^ and RhoA^fl/fl^LckCre^−^ mice, respectively (Figure 1A). Furthermore, among the RhoA^fl/fl^LckCre^+^ mice that developed EAE (75%), day of disease debut was significantly delayed by 15–30 days in these mice compared with RhoA^fl/−^LckCre^+^ and RhoA^fl/fl^LckCre^−^ mice (Figure 1B). EAE in RhoA^fl/fl^LckCre^+^ mice was characterized by a lower clinical score and relapse-remission frequency compared with RhoA^fl/−^LckCre^+^ or RhoA^fl/fl^LckCre^−^ mice (Figure 1C; Figures S3A–C and Table S1 in Supplementary Material). Moreover, the cumulative EAE score, i.e., overall EAE disease severity from day of disease debut until day 37, showed significantly less disease activity in the RhoA^fl/fl^LckCre^+^ mice (Figure 1D). In addition, maximum EAE score (Figure 1E) and mean EAE score in the acute phase (Figure 1F) also showed significantly less disease severity in the RhoA^fl/fl^LckCre^+^ mice. No significant differences were observed in the EAE disease severity between RhoA^fl/fl^LckCre^−^ and RhoA^fl/−^LckCre^+^ mice. These results strongly suggest that the lack of RhoA significantly reduces the capacity of T-cells to induce EAE and sustain disease severity.

### Lack of RhoA Expression Halts T-Cell Infiltration in Brain Parenchyma

Given the less severe EAE disease pattern observed in the RhoA^fl/fl^LckCre^+^ mice, we next wanted to investigate whether lack of RhoA affected the T-cells’ ability to enter the CNS and induce disease. We chose to exclude RhoA^fl/+^LckCre^+^ mice in all subsequent analyses as the EAE disease pattern in those mice did not differ significantly from RhoA^fl/fl^LckCre^−^ mice.

**Figure 2:**
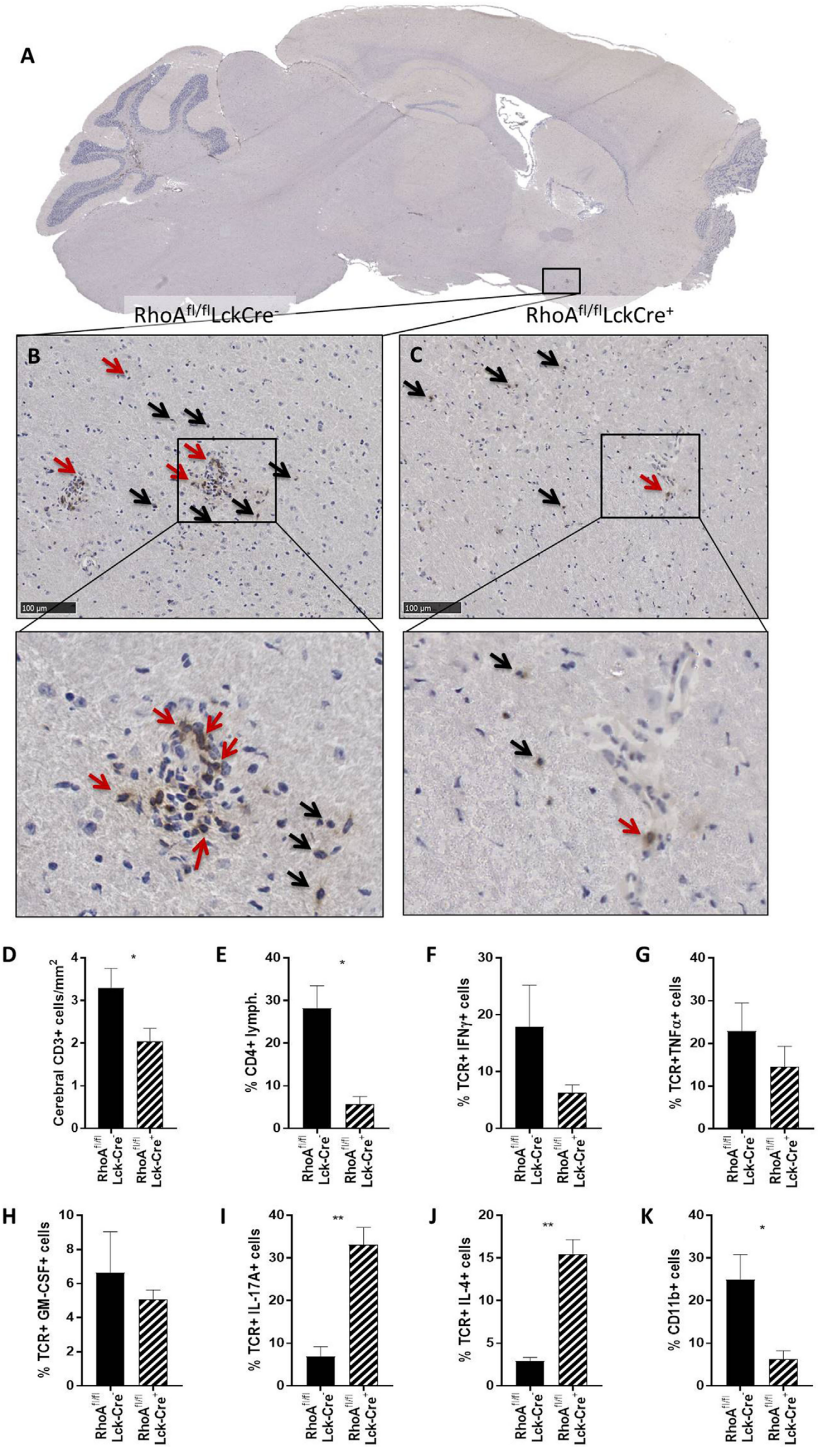
Lack of RhoA expression prevents T-cell infiltration in brain parenchyma. **(A)** Representative micrograph of a sagittal brain section from a RhoA^fl/fl^LckCre^−^ mouse, stained for CD3. Magnification of the brain section from a RhoA^fl/fl^LckCre^−^ mouse **(B)** and a RhoA^fl/fl^LckCre^+^ mouse **(C)**, where CD3^+^ T-cells can be observed in the brain parenchyma (black arrows) and the perivascular space (red arrows). **(D)** Quantification by IHC of the CD3^+^ T-cells present in the cerebral area of the brain section from RhoA^fl/fl^LckCre^−^ (*n* = 33) and RhoA^fl/fl^LckCre^+^ (*n* = 24) brain sections. Data are represented as number of CD3^+^ T-cells per area (mm^2^). **(E–K)** Quantification by flow cytometry of CD4^+^ T-cells **(E)**, IFNγ^+^ T-cells **(F)**, TNFα^+^ T-cells **(G)**, GM-CSF^+^ T-cells **(H)**, IL-17A^+^ T-cells **(I)**, IL-4^+^ T-cells **(J)**, and CD11b^+^ cells **(K)** present in spinal cord of experimental autoimmune encephalomyelitis mice (*n* = 3 per group). Data shown as mean ± SEM. *p < 0.05, **p < 0.01 (unpaired *t*-test).

We first quantified the number of CD3^+^ T-cells present in four brain sections per mouse of each genotype in the cerebral parenchyma, PVS as well as meninges and ventricular system (from here on referred to as meninges/ventricles) (Figures 2A–C). We observed no significant differences in the number of CD3^+^ T-cells present in the whole brain of RhoA^fl/fl^LckCre^−^ and RhoA^fl/fl^LckCre^+^ mice (Figure S4A in Supplementary Material). However, investigating the capacity of T-cells to migrate through parenchyma, RhoA^fl/fl^LckCre^+^ mice presented significantly less CD3^+^ T-cells in the cerebral parenchyma compared with RhoA^fl/fl^LckCre^−^ mice (Figure 2D). To examine whether RhoA^−/−^ T-cells were trapped in the meninges/ventricles or in the PVS, we quantified the CD3^+^ T-cells present in these regions. We did not detect significant differences in the number of CD3^+^ T-cells in these regions between RhoA^fl/fl^LckCre^−^ and RhoA^fl/fl^LckCre^+^ mice (Figures S4B,C in Supplementary Material).

Immune cell infiltration in spinal cord highly correlates with EAE pathogenesis, thus we analyzed infiltrating cells and cytokine production in spinal cords of RhoA^fl/fl^LckCre^+^ and RhoA^fl/fl^LckCre^−^ mice by flow cytometry. Similar to the immunohistochemical count of total brain CD3^+^ cells (Figure S4A in Supplementary Material), the analysis revealed no differences in the total spinal cord infiltrating lymphocytes (Figure S4D in Supplementary Material). However, RhoA^fl/fl^LckCre^+^ mice presented significantly reduced numbers of CD4^+^ cells in spinal cord compared with RhoA^fl/fl^LckCre^−^ mice (Figure 2E). Furthermore, CNS infiltrated T-cells in RhoA^fl/fl^LckCre^+^ mice produced reduced amounts of the pro-inflammatory cytokines IFNγ (Figure 2F), TNFα (Figure 2G), and GM-CSF (Figure 2H), although not statistically significant. Surprisingly, we found significantly elevated levels of the Th17 cytokine IL-17A (Figure 2I) and the Th2 cytokine IL-4 (Figure 2J) in these cells. Presence of infiltrating CD11b^+^ macrophages was also reduced in spinal cords of RhoA^fl/fl^LckCre^+^ mice compared with RhoA^fl/fl^LckCre^−^ mice (Figure 2K). These results indicate that lack of RhoA compromises T-cell’s ability to infiltrate the CNS and mount a Th1 pro-inflammatory response. The reduction of infiltrating macrophages in RhoA^fl/fl^LckCre^+^ mice is probably a consequence of the reduced pro-inflammatory profile of T-cells, which results in reduced BBB breakdown, reduced immune cell infiltration, and reduced APC activation.

### Lack of RhoA Prevents MOG-Specific Effector T-Cell Responses

Although in support of lower EAE disease, we found less CD3^+^ T-cells in the cerebral parenchyma and less CD4^+^ T-cells in spinal cord of RhoA^fl/fl^LckCre^+^ mice compared with RhoA^fl/fl^LckCre^−^ mice, the unexpected findings that the number of CD3^+^ T-cells were similar in the meninges, ventricular system, and PVSs could be an indication that RhoA might play an essential role in antigen-specific T-cell activation, proliferation, and/or cytokine production in addition to T-cell migration. To address the role of RhoA in T-cell activation, we investigated whether the decreased disease activity in RhoA^fl/fl^LckCre^+^ mice could be due to defects in antigen-specific, MOG_35–55_ T-cell responses.

**Figure 3:**
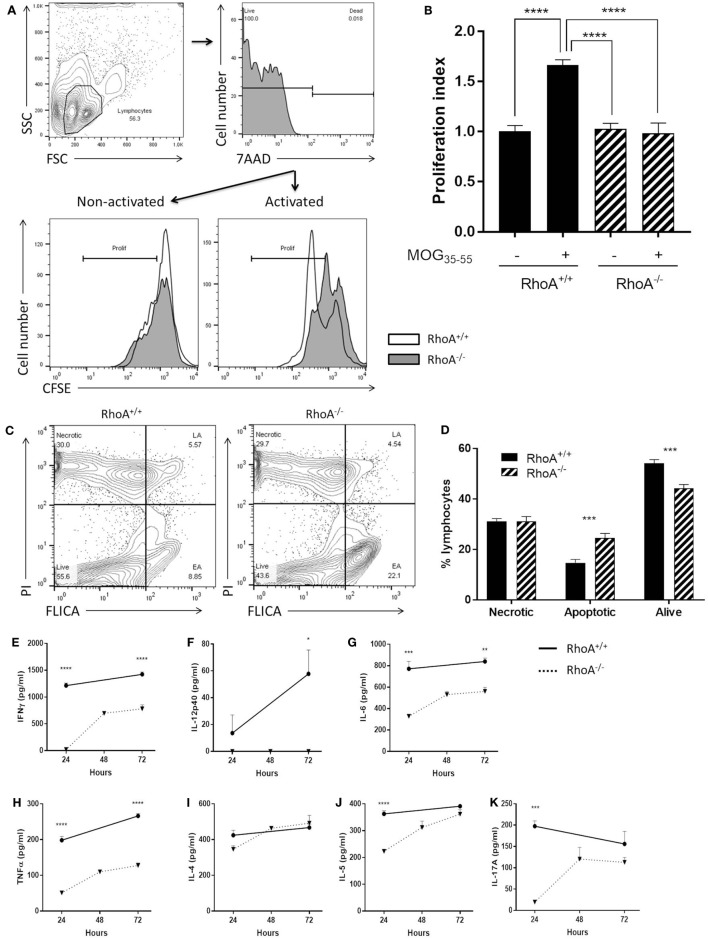
Lack of RhoA halts T-cell immune responses against the encephalitogenic antigen, MOG_35–55_. **(A)** Representative histogram of the proliferation of RhoA^+/+^ and RhoA^−/−^ T-cells *in vitro* based on their CFSE labeling. **(B)** Quantification of the proliferation index (fold increase in proliferation in relation to unstimulated RhoA^+/+^ T-cells) of RhoA^+/+^ (filled bars) and RhoA^−/−^ (striped bars) T-cells upon MOG_35–55_ activation for 48 h. Data represents the mean of two independent experiments (RhoA^+/+^ *n* = 4, RhoA^−/−^ *n* = 3). **(C)** Representative gating strategy for the analysis of cell viability. **(D)** Quantification of the percentage of necrotic (N), apoptotic (EA + LA) and living (L) cells in RhoA^+/+^ and RhoA^−/−^ T-cells after 24 h of MOG_35–55_-activation *in vitro*. **(E–K)** Quantification of the amount of IFN-γ, IL-12p40, IL-6, TNF-α, IL-4, IL-5, and IL-17A present in the supernatant of RhoA^+/+^ (continuous line) and RhoA^−/−^ (dotted line) T-cells after 24, 48, and 72 h in culture in the presence of MOG_35–55_. Data shown as mean ± SEM *p < 0.05, **p < 0.01, ***p < 0.001, ****p < 0.0001 [**(B)** One-way ANOVA, followed by Tukey’s test. **(D)** Multiple *t*-test, followed by FDR correction. **(E–J)** Multiple *t*-test, followed by FDR correction]. Abbreviation: FSC, forward scatter; SSC, side scatter; PI, propidium iodide; FLICA, fluorescent inhibitor of caspases; L, living; N, necrotic; EA, early apoptotic; LA, late apoptotic; FDR, false discovery rate.

We investigated the proliferative capacity of T-cells in response to MOG_35–55_ by studying T-cells purified from MOG_35–55_-immunized RhoA^fl/fl^LckCre^−^ compared with RhoA^fl/fl^LckCre^+^ mice, i.e., RhoA^+/+^ and RhoA^−/−^ T-cells, based on CFSE labeling and flow cytometric detection. Lack of RhoA expression significantly compromised the T-cell’s ability to proliferate in response to the antigen-specific stimulation (Figures 3A,B). Interestingly, further analysis revealed that poor MOG_35–55_ response in RhoA^−/−^ T-cells is associated with lack of survival, as MOG_35–55_ activation of RhoA^−/−^ T-cells results in significantly elevated rates of apoptosis (Figures 3C,D). These results indicate that MOG_35–55_ immunization of mice generates a poor immune response when T-cells lack RhoA compared with RhoA competent T-cells.

Next, it was of interest to analyze if poor ability of RhoA^−/−^ T-cells to proliferate in response to MOG_35–55_ could also impact their cytokine profile, which is closely associated with antigen-specific T-cell capacity to induce EAE (1, 6). We therefore quantified an array of Th1/Th2/Th17 prototype cytokines produced in the culture supernatant of RhoA^+/+^ and RhoA^−/−^ T-cells after 24, 48, and 72 h of stimulation with MOG_35–55_. RhoA^−/−^ T-cells produced significantly less Th1 pro-inflammatory cytokines, as evidenced by the low expression of IFN-γ and the complete lack of IL-12 production (Figures 3E,F). Production of the pro-inflammatory cytokines IL-6 and TNF-α, often associated with Th1 responses and disease activity, were also significantly reduced in MOG_35–55_ stimulated RhoA^−/−^ T-cells compared with the RhoA^+/+^ T-cells (Figures 3G,H). Regarding the Th2 and Th17 cytokines, even though RhoA^−/−^ T-cells can produce similar amounts of cytokines after 72 h of MOG_35–55_ stimulation, their production is reduced in early time points, which is significantly pronounced in the case of IL-5 and IL-17A (Figures 3I–K).

Taken together, these results indicate that lack of RhoA significantly compromises the T-cells’ ability to respond to their cognate antigen and are thereby defective in inducing EAE.

### RhoA Is Important for Antigen-Independent T-Cell Responses

After firmly having established that effector T-cells isolated from MOG-immunized RhoA^fl/fl^LckCre^+^ mice are unable to mount a proper recall antigen response, we wanted to test whether this was unique to effector RhoA^−/−^ T-cell responses, or whether naïve RhoA^−/−^ T-cells would exhibit the same defects when activated with anti-CD3/anti-CD28 antibodies.

**Figure 4:**
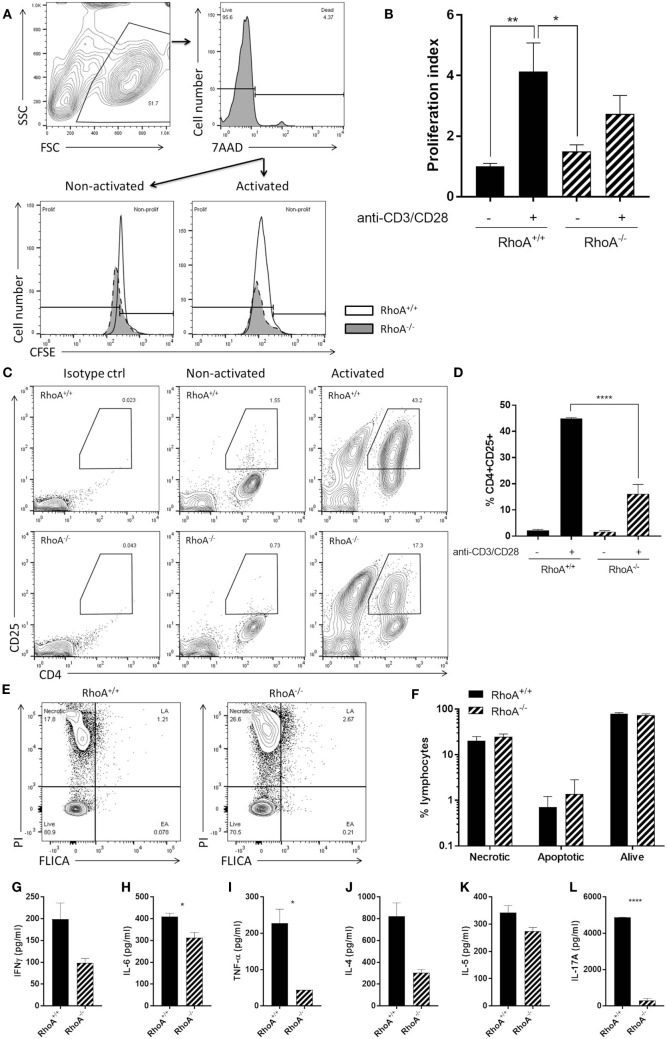
RhoA expression is important for pan T-cell responses. **(A)** Representative histogram of the proliferation of RhoA^+/+^ and RhoA^−/−^ T-cells *in vitro* based on their CFSE labeling. **(B)** Proliferation index of RhoA^+/+^ (filled bars) and RhoA^−/−^ (striped bars) T-cells upon pan-activation with anti-CD3/CD28 antibodies for 72 h. Data represent the mean of two independent experiments, *n* = 3 mice per group. **(C)** Representative gating strategy for the analysis of cell activation. **(D)** Quantification of the percentage of CD4^+^CD25^+^RhoA^+/+^ and RhoA^−/−^ T-cells in the presence or absence of pan-activation. Data represent the mean of two independent experiments, *n* = 3 mice per group. **(E)** Representative gating strategy for the analysis of cell viability. **(F)** Quantification of the percentage of necrotic (N), apoptotic (EA + LA) and living (L) cells in RhoA^+/+^ and RhoA^−/−^ T-cells after 24 h of pan-activation *in vitro*. Data represent the mean of three independent experiments, *n* = 4 mice per group. **(G–L)** Amount of IFN-γ, IL-6, TNF-α, IL-4, IL-5, and IL-17A present in the supernatant of RhoA^+/+^ and RhoA^−/−^ T-cells after 24 h stimulation with anti-CD3/CD28. Data shown as mean ± SEM *p < 0.05, **p < 0.01, ***p < 0.001, ****p < 0.0001 [**(B)** Two-way ANOVA, followed by Tukey’s test. **(D)** One-way ANOVA, followed by Tukey’s test. **(F)** Multiple *t*-test, followed by FDR correction. **(G–K)** Unpaired *t*-test]. Abbreviation: FSC, forward scatter; SSC, side scatter; PI, propidium iodide; FLICA, fluorescent inhibitor of caspases; L, living; N, necrotic; EA, early apoptotic; LA, late apoptotic; FDR, false discovery rate.

Similar to what we observed in the effector T-cell population, RhoA^−/−^ T-cells presented a significantly lower proliferative capacity compared with RhoA^+/+^ T-cells (Figures 4A,B). By quantifying the surface levels of the activation marker CD25 of splenic CD4^+^ T-cells isolated from RhoA^fl/fl^LckCre^−^ and RhoA^fl/fl^LckCre^+^ mice (Figure 4C), we observed that RhoA^−/−^ T-cells reach a significantly lower (~60% less) activation state compared with RhoA^+/+^ T-cells in response to activation with anti-CD3/anti-CD28 antibodies (Figure 4D).

When studying the viability of RhoA^+/+^ and RhoA^−/−^ T-cells, based on their labeling intensity of PI and FLICA (Figure 4E), we observed no differences in the frequency of apoptotic, necrotic, or alive RhoA^−/−^ T-cells compared with RhoA^+/+^ T-cells, although RhoA^−/−^ T-cells showed a tendency toward more apoptosis (Figure 4F). This resembles the higher apoptosis rate observed in MOG_35–55_-specific effector/memory RhoA^−/−^ T-cells (Figure 3D).

Upon anti-CD3/anti-CD28 stimulation, similar to MOG_35–55_-specific stimulation, the cytokine production of T-cells was significantly decreased in the absence of RhoA. RhoA^−/−^ T-cells present a tendency to a lower production of the classical Th1 cytokine IFN-γ (Figure 4G) and a significantly reduced production of the pro-inflammatory Th1-associated cytokines IL-6 and TNF-α (Figures 4H,I). In this case, the Th1 cytokine IL-12 was not detectable in either RhoA^+/+^ or RhoA^−/−^ T-cells (data not shown). We also observed a trend to a reduced production of Th2 cytokines IL-4 and IL-5 by RhoA^−/−^ T-cells after 24 h of anti-CD3/anti-CD28 stimulation (Figures 4J,K), which resembles our observations at the 24-h time point with MOG_35–55_-specific effector/memory T-cells (Figures 3I,J). RhoA^−/−^ T-cells also presented a significantly reduced production of the Th17 cytokine IL-17A (Figure 4L). Taken together, these results suggest that RhoA is important for both the initial activation of naïve T-cells and reactivation of effector T-cells.

### RhoA Expression Is Crucial for T-Cell Transendothelial Migration

**Figure 5:**
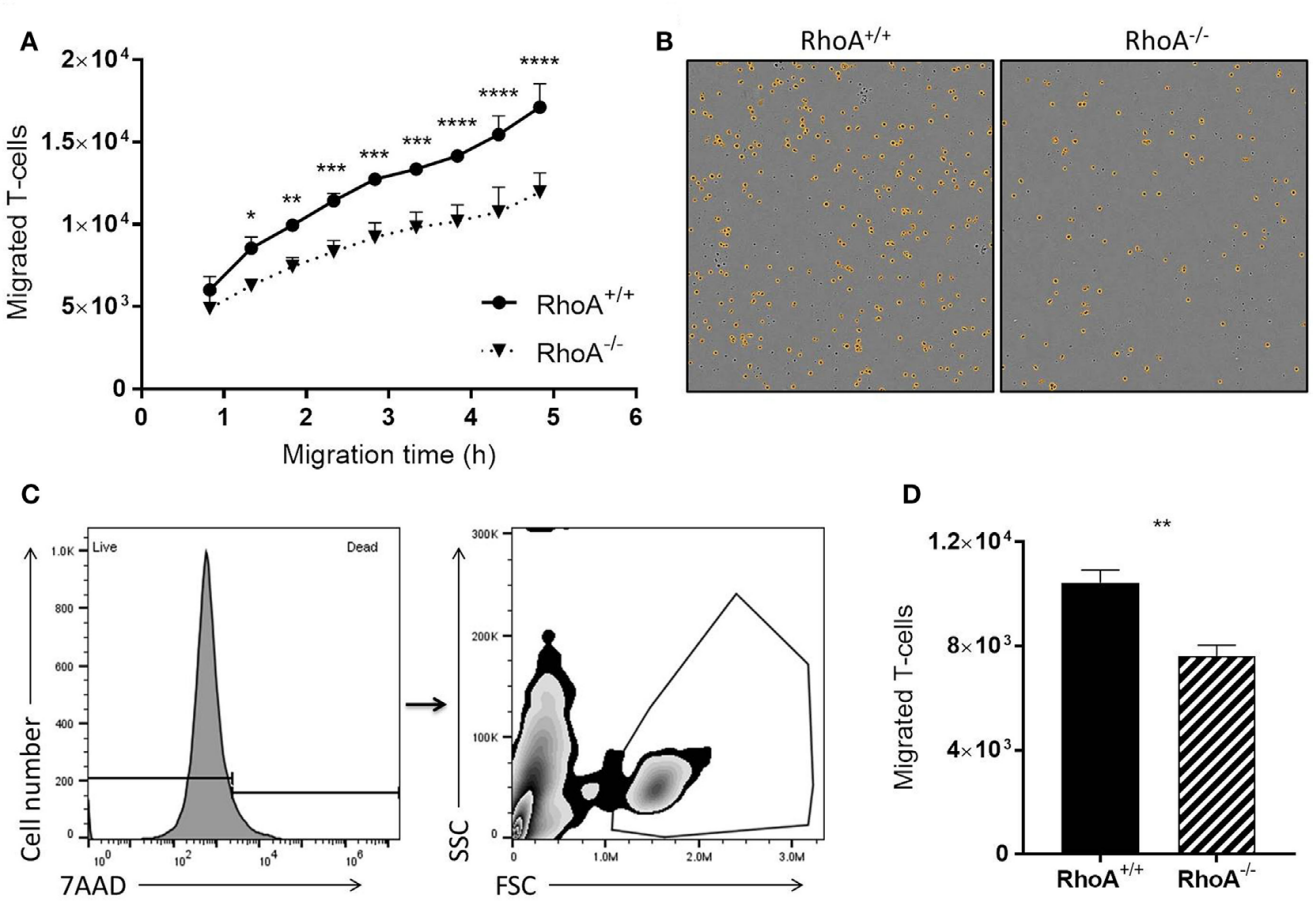
RhoA is important in T-cell transendothelial migration. **(A)** Quantification of migratory capacity of RhoA^+/+^ and RhoA^−/−^ T-cells across an endothelium, analyzed by live-cell time lapse imaging. Data representative of two independent experiments. **(B)** Representative images of migrated RhoA^+/+^ (left) and RhoA^−/−^ (right) T-cells at the bottom of the migration chamber after 4 h of migration. Marked in orange are the migrated cells detected and quantified by the software IncuCyte™ Basic Software **(C)**. Gating strategy used for flow cytometric analysis of migrated T-cells. **(D)** Quantification of the total number of migrated cells after 4 h by flow cytometry. Data represent the mean of two independent experiments. Data shown as mean ± SEM *p < 0.05, **p < 0.01, ***p < 0.001, ****p < 0.0001 [**(A)** Two-way ANOVA, followed by Sidak’s test. **(D)** Unpaired *t*-test].

It is well established that RhoA is a main regulator of cellular migration (14, 29). We therefore proceeded to investigate if the migratory capacity of RhoA^−/−^ T-cells is also compromised, which could contribute to the manifested significantly lower clinical EAE. We established a confluent layer of bEnd.3 cells to mimic BBB and measured ability of T-cells to cross this endothelial cell barrier. As shown in Figure 5A, RhoA^−/−^ T-cells migrate significantly slower across the endothelial barrier compared with RhoA^+/+^ T-cells, resulting in ~27% fewer cells after 4 h of migration (Figures 5B–D). This indicates that RhoA plays an important role in the migratory capacity of T-cells across an endothelial barrier like the BBB.

## Discussion

Although RhoA signaling has been reported to be important for T-cell activation *in vivo* and in various T-cell migration *in vitro* models (14), the direct impact of RhoA on T-cell’s capacity to cross BBB and induce disease in the CNS has not been established. In this study, using mice with a T-cell-specific RhoA gene deletion (RhoA^fl/fl^LckCre^+^ mice) we show that RhoA is important in various aspects of T-cell biology as well as for the T-cell’s ability to induce severe EAE.

T-cell infiltration into the CNS is tightly linked to the pathogenesis of EAE and MS (2, 3, 6, 30). In turn, T-cell migration is dependent on cytoskeletal rearrangements, which are regulated by Rho GTPases (14). Previous studies have shown that the treatment of splenic mononuclear cells (MNCs) with fasudil, which inhibits the Rho downstream kinase ROCK (31, 32), ameliorates the clinical severity of active and adoptive EAE and reduces MNCs’ CNS infiltration (23). The researchers suggest that fasudil treatment reduces the encephalitogenicity of MNCs and induces macrophage polarization from pathogenic M1 to beneficial M2 macrophages (23). However, ROCK inhibition with fasudil inhibits signaling from GTPases other than RhoA, i.e., RhoB and RhoC, and treating mice systemically with fasudil could generally impact ROCK signaling in all types of cells expressing ROCK, hence making it difficult to address the specific role of RhoA in T-cells only. Here, we show for the first time that T-cell specific deletion of RhoA is enough to ameliorate EAE severity. Our results show that the lack of RhoA in T-cells markedly delays the EAE onset and reduces the EAE incidence and clinical severity. The histopathological analysis of CNS tissue from EAE mice revealed significantly reduced numbers of infiltrating T-cells in the cerebral parenchyma and spinal cords of RhoA^fl/fl^LckCre^+^ mice compared with RhoA^fl/fl^LckCre^−^ mice. Furthermore, CNS-infiltrating T-cells of RhoA^fl/fl^LckCre^+^ mice showed reduced production of the pro-inflammatory cytokines IFNγ, TNFα, and GM-CSF in spinal cord, tightly linked to EAE pathogenesis and disease activity. Interestingly, we also observed a significantly reduced infiltration of CD11b^+^ cells in the spinal cord of RhoA^fl/fl^LckCre^+^ mice, which could be a consequence of lower levels of T cell activation and pro-inflammatory cytokine production in the periphery to impact directly APC activation and APC recruitment, or indirectly by reduced BBB breakdown and hence lower infiltration in the area. These criteria are also important reasons behind the ameliorated EAE observed in RhoA^fl/fl^LckCre^+^ mice, given that EAE severity is tightly related to the level of T-cell and macrophage infiltrates in the CNS.

The small GTPase RhoA regulates a range of T-cell functions including development, activation, cell cycle, proliferation, and migration (14, 33, 34). Studies have shown that inhibition of Rho signaling with C3 transferase results in reduced thymic cellularity and reduced numbers of mature T-cells in the periphery [96, 97, 108]. It has also been demonstrated that Rho signaling is present in the immunological synapse and downstream of the T-cell receptor (TCR) (35), and RhoA is known to be involved in TCR-mediated signal transduction (36). Moreover, T-cells expressing a constitutively active RhoA (V14RhoA) have been shown to be hyperresponsive in the context of TCR-induced proliferation (37), and a study using mice knock-out for TAGAP, a T-cell-specific RhoA-activating protein, demonstrated that RhoA activation enhances TCR-mediated Th17 differentiation, thereby contributing to EAE pathogenesis (38). Consistent with these findings, we show that RhoA^fl/fl^LckCre^+^ mice present reduced numbers of mature T-cells in thymus and spleen but normal counts in peripheral blood. Furthermore, lack of RhoA reduces T-cell activation capacity, increases T-cell apoptosis rates, and significantly reduces proliferation of both autoreactive effector/memory T-cells and naïve T-cells. It is known that reactivation of T-cells by macrophages in the CNS parenchyma is important during CNS invasion and inflammation in MS and EAE (39). The impaired activation capacity of RhoA-deficient T-cells and the decreased number of macrophages in the CNS of RhoA^fl/fl^LckCre^+^ mice might result in defective T-cell reactivation, further contributing to the decreased EAE severity observed in these mice. The reduced T-cell activation capacity of RhoA-deficient T-cells is most likely the cause of their reduced pro-inflammatory cytokine production. The impaired T-cell development together with the reduced activation and proliferative capacity and the elevated cell death might be underlying causes of the reduced numbers of T-cells present in the CNS after EAE in RhoA^fl/fl^LckCre^+^ mice.

Cytokines are important mediators of T-cell function and pathogenicity, and they are of interest as therapeutic targets for inflammatory diseases like MS (40). Indeed, the immunomodulators IFN-β and Glatiramer acetate, which constitute the first-line treatment for MS, downregulate the production of chemokines and pro-inflammatory cytokines such as IFN-γ and IL-6 (41, 42). Supporting pro-inflammatory cytokine-signaling pathways as targets of therapy, the anti-IL2Rα antibody Daclizumab has been approved for the treatment of RRMS (43), and the blockage of the GM-CSF receptor has been shown beneficial in a RR-EAE mouse model (44). Antibody therapy with Ustekinumab, which targets the cytokines IL-12/23, has been shown to inhibit EAE in mice (45), even though it failed to show efficacy in RRMS patients, supposedly due to low CNS availability of the compound or to the advanced stage of the RRMS pathology at the time of treatment (46). Here, we show that the lack of RhoA reduces the pro-inflammatory cytokines’ production by both autoreactive effector/memory T-cells and naïve T-cells *in vitro*, indicating a dampened pathogenic T-cell reactivity in the absence of RhoA. Similarly, CNS infiltrated RhoA^−/−^ autoreactive T-cells produced reduced amounts of the pro-inflammatory cytokines IFNγ, TNFα, and GM-CSF in comparison to RhoA^+/+^ T-cells. Interestingly, and contrarily to our observations *in vitro*, we found significantly more IL-17- and IL-4-producing T-cells in the CNS of RhoA^fl/fl^LckCre^+^ mice compared with RhoA^fl/fl^LckCre^−^ mice. The fact that a different and more complex inflammatory milieu exists *in vivo* could explain these discrepancies. Furthermore, this could illustrate a change in the T-cell phenotype as the disease progresses, changing from a Th1 phenotype to a Th17 and Th2 phenotype. In addition, the T-cells were isolated from inflamed spinal cords at different stages of disease progression, given the significantly later disease debut in the RhoA^fl/fl^LckCre^+^ mice.

Given the key regulatory role of Rho GTPases in cell migration (47), there has been effort to inhibit Rho GTPases to inhibit lymphocyte recruitment to the CNS (48). Our current study strongly supports that the lack of RhoA specifically in T-cells reduces their migratory capacity and velocity compared with RhoA^+/+^ T-cells, associated with lower incidence and severity of disease in RhoA^fl/fl^LckCre^+^ mice. The proof of concept that inhibiting the recruitment of pathogenic T-cells to the CNS is beneficial for MS has been demonstrated in MS patients treated by Natalizumab, a humanized monoclonal antibody reacting against the integrin very late antigen 4, thereby inhibiting lymphocyte migration (9).

Collectively, our results demonstrate the importance of RhoA in T-cells in the initiation and development of EAE, which is linked to a reduced response to external stimuli resulting in a reduction in CNS inflammation and reduced disease activity in a model for MS. Some of the currently approved disease-modifying therapies for MS target T-cell migration (Natalizumab, Fingolimod), cytokine secretion (IFN-β, Glatiramer acetate), and T-cell proliferation (Teriflunomide, Mitoxantrone) (49). Interestingly, as shown here, RhoA plays a role in all of these central T-cell processes, also essential for their capacity to induce neuroinflammation. Thus, our current study also highlights the therapeutic potential of RhoA as an interesting target to reduce CNS T-cell infiltration and thereby inflammation in MS.

## Ethics Statement

All procedures were approved and performed in accordance with the Danish Animal Experimentation Committee (#2012-DY-2934-00001) and the European Council Directive #86/609 for the Care of Laboratory Animals.

## Author Contributions

AM-A and HH contributed to the conception and design of the work. AM-A contributed to the acquisition, analysis, and interpretation of the data. SI-N and HH contributed to the analysis and interpretation of data for the work. AM-A, SI-N, and HH contributed to the drafting of the work. AM-A, FJ, CB, SI-N, and HH revised the work critically for important intellectual content and gave the final approval of the revision to be published.

## Conflict of Interest Statement

The research was conducted in the absence of any commercial or financial relationships that could be construed as a potential conflict of interest.
