# Supplementary material for: RhoA Drives T-Cell Activation and Encephalitogenic Potential in an Animal Model of Multiple Sclerosis
Source: Front Immunol. 2018 May 31;9:1235. doi: 10.3389/fimmu.2018.01235 (PMC5990621; doi:10.3389/fimmu.2018.01235)
Supplement: Supplementary file 1 [file Data_Sheet_1.DOCX]

Supplementary Material

**RhoA drives T-cell activation and encephalitogenic potential in an animal model of Multiple Sclerosis**

**Alba Manresa-Arraut^1^, Flemming Fryd Johansen^1^, Cord Brakebusch^2^, Shohreh Issazadeh-Navikas^1^, Henrik Hasseldam^1*^**

*** Correspondence:** Henrik Hasseldam: henrik.hasseldam@bric.ku.dk

# Supplementary Figures and Tables

## Supplementary Table 1: RhoA is important for the relapsing/remitting frequency of EAE

## Supplementary Table 1: RhoA^fl/fl^LckCre^+^ mice present significantly reduced relapse/remission frequency compared to RhoA^fl/fl^LckCre^-^ mice.

| Group | Relapse frequency | Remission frequency |
| --- | --- | --- |
| RhoA^fl/fl^LckCre^-^ | 12/28 = 42.85% | 27/28 = 96.43% |
| RhoA^fl/+^LckCre^+^ | 2/10 = 20% ** | 10/10 = 100% |
| RhoA^fl/fl^LckCre^+^ | 2/10 = 20% ** | 6/10 = 60% **** |

Relapsing frequency: Number of relapsing mice is calculated from total number of mice.

Remitting frequency: Number of remitting mice is calculated from total number of mice.

**p=0.001, **** p<0.0001 *Chi*-square test when compared to RhoA^fl/fl^LckCre^-^ mice, indicating that RhoA^fl/fl^LckCre^-^ mice present significantly higher relapsing frequency compared to RhoA^fl/+^LckCre^+^ and RhoA^fl/fl^LckCre^+^ mice and a higher remitting frequency compared to RhoA^fl/fl^LckCre^+^ mice.

## Supplementary Figures

(see JPEG of the figures uploaded as a separate files)

### Supplementary figure 1: Generation of mice with a T-cell-restricted deletion of the RhoA gene.

**A.** Representative fluorescent immunocytochemistry micrographs of RhoA expression in RhoA^+/+^ and RhoA^-/-^ T-cells. **B.** RhoA staining intensity presented as integrated density (IntDen) in each cell (RhoA^+/+^ n=39, RhoA^-/-^ n=31). Data shown as mean±SEM **p<0,05, **p<0,01, ***p<0,001, **** p<0,0001 (unpaired t-test).

### Supplementary figure 2: RhoA is important for T-cell development and maturation.

A. Representative gating strategy from thymus for analysis of the different cell populations. The same gating strategy was used for the analysis of spleen and blood. B-S. Quantification of the populations of lymphocytes, CD3^+^, CD4^+^, CD8^+^ and CD4^+^CD8^+^ T-cells in blood (B-G), thymus (H-M) and spleen (N-S) of naïve 129Sv/C57BL/6 mice RhoA^fl/fl^LckCre^-^ (n=16), RhoA^fl/+^LckCre^+^ (n=6) and RhoA^fl/fl^LckCre^+^ (n=10). Data shown as mean±SEM *p<0,05, **p<0,01 (two-way ANOVA, followed by Tukey’s test).

### Supplementary figure 3: Lack of RhoA reduces EAE disease severity.

The data show 5 representative mice for each genotype: RhoA^fl/fl^LckCre^-^ (A) RhoA^fl/+^LckCre^+^ (B) and RhoA^fl/fl^LckCre^+^ (C). Red line represents the mean EAE score of all mice in each genotype (RhoA^fl/fl^LckCre^-^ n=39, RhoA^fl/+^LckCre^+^ n=13, RhoA^fl/fl^LckCre^+^ n=12).

### Supplementary figure 4: Quantification of CNS infiltrating immune cells in EAE mice.

A-C. Histopathological quantification of the CD3^+^ T-cells present in the total area of the brain section (A), meninges/ventricles (B) and PVS (C) from RhoA^fl/fl^LckCre^-^ (n=33) and RhoA^fl/fl^LckCre^+^ (n=24) brain sections. PVS – perivascular space. Data is represented as number of CD3^+^ T-cells per area (mm2). D. Quantification by flow cytometry of lymphocytes present in spinal cords of EAE mice (n=3 per group). Data shown as mean±SEM. *p<0,05 (unpaired t-test).

## Supplementary Materials and Methods:

### RhoA quantification

Spleens were isolated from naïve RhoA^fl/fl^LckCre^-^ and RhoA^fl/fl^LckCre^+^ mice and single-cell suspensions were prepared by mechanical disruption through 40 µm cell strainers. Naïve CD3^+^ cells were enriched using a MACS Pan T-cell isolation kit II mouse (Miltenyi Biotec) and cells were prepared for immunocytochemical staining. Briefly, 5x10^5^ cells were in 50µl of cell staining buffer (Biolegend) were incubated with the permeabilization/fixation solution (eBioscience) for 30min at RT. Cells were subsequently washed, resuspended in 50µl of cell staining buffer, stained with anti-mouse TCR FITC (2µl/test, BD Pharmingen clone H57-597) and anti-mouse RhoA PE (5µl/test, Santa Cruz clone 26C4) for 30min at RT. After washing wells were transferred to collagen pre-coated slides and let adhere for 1h at RT. Excess of buffer was aspirated and the cells were mounted with Vectashield™ mounting medium containing DAPI (Vectorlabs, Burlingame, USA) for nuclei staining. Slides were visualized under a Zeiss fluorescence microscope and pictures of 30 to 40 cells were taken from each genotype. Quantification of RhoA expression in each cell was done by in ImageJ using standard thresholding and integrated density measurements.

### Tissue preparation

Spleen, thymus and intra-cardiac blood were collected from 12 to 21 week-old naïve female 129Sv/C57Bl/6 mice (RhoA^fl/fl^LckCre^-^ n=16, RhoA^fl/+^LckCre^+^ n=6 and RhoA^fl/fl^LckCre^+^ n=10). Mice were anesthetized with 5% isoflurane (Forene) delivered in pure oxygen and blood was extracted from the left ventricle of the heart and transferred immediately into a EDTA-coated tubes. Mice were perfused transcardially with PBS until blood was cleared from the circulation, and spleen and thymus were collected. Blood was lysed in red blood cell lysis buffer (eBioscience) for 5 minutes at room temperature prior preparation for flow cytometry analysis. Single cell suspensions of spleen and thymus were prepared by mechanical disruption and subsequently prepared for flow cytometry analysis.

### Flow cytometry analysis of the T-cell pool in naïve mice

Briefly, 5x10^5^cells in 50µl of cell staining buffer (Biolegend) were incubated with the viability marker Zombie UV™ (Biolegend) for 20min at RT. Cells were subsequently washed, resuspended in 50µl of cell staining buffer, stained with American hamster anti-mouse CD3ε FITC (2µl/test, Biolegend clone 145-2C11), rat anti-mouse CD4 PE-Cy7 (1µl/ml, BioLegend clone GK1.5) and rat anti-mouse CD8b.2 APC (1µl/test, Biolegend clone 53-5.8) or isotype controls for 30min on ice. Cells were then washed, resuspended in 400µl of cell staining buffer and analysed by flow cytometry. Flow cytometry was performed using the LSRII (BD Biosciences) and the CellQuest software (BD Biosciences). Data analysis was done using the FlowJo V10 software (Tree Star). Isotype controls were included as negative controls and subtracted.
