# Supplementary figures and images for: RhoA Drives T-Cell Activation and Encephalitogenic Potential in an Animal Model of Multiple Sclerosis
Source: Front Immunol. 2018 May 31;9:1235. doi: 10.3389/fimmu.2018.01235 (PMC5990621; doi:10.3389/fimmu.2018.01235)

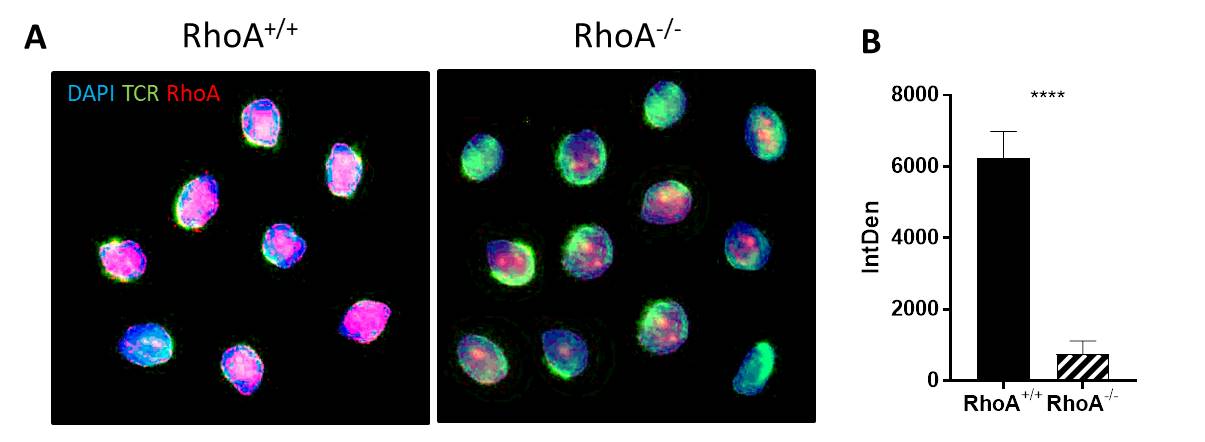

Supplement: Supplementary file 2 [file Image_1.JPEG]

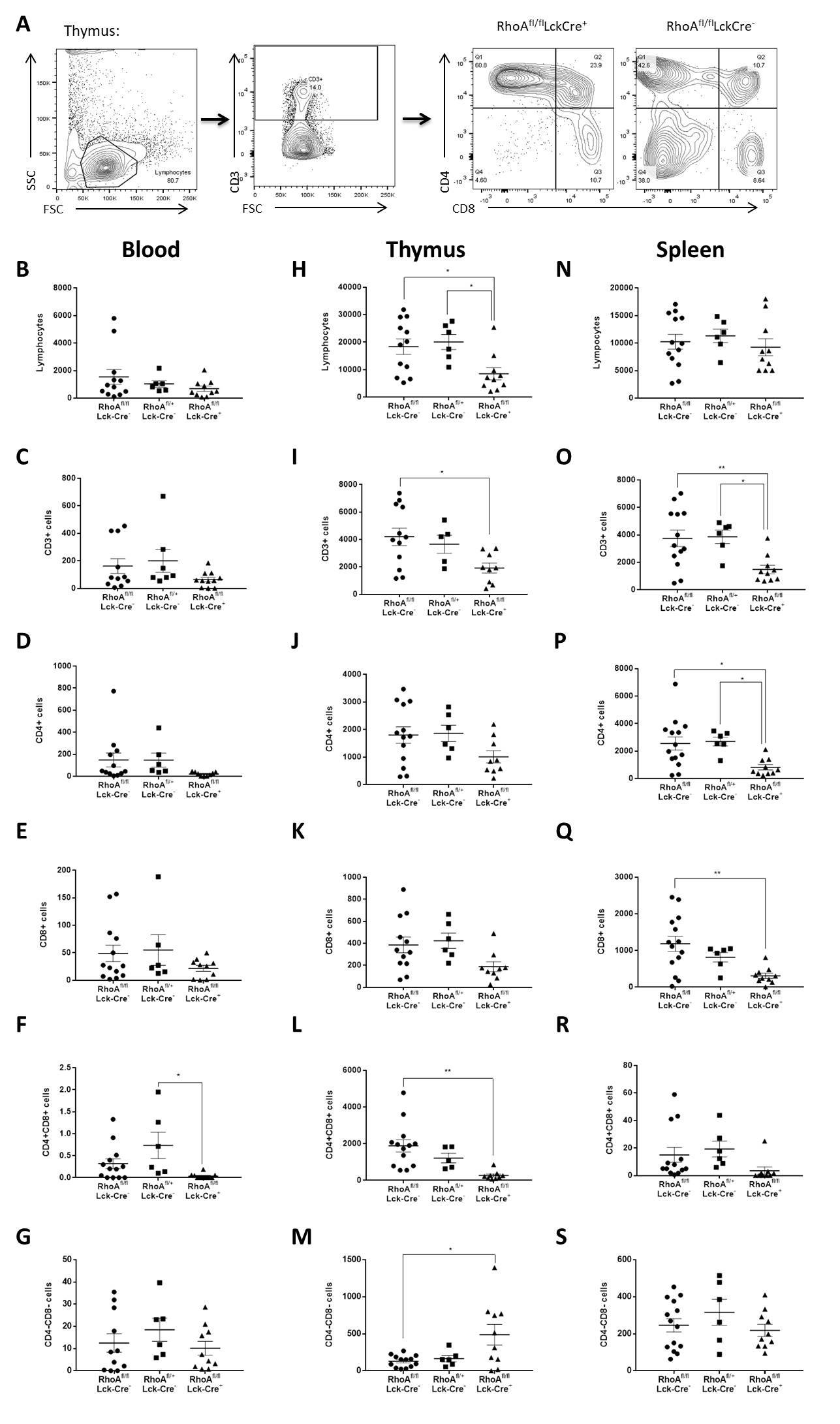

Supplement: Supplementary file 3 [file Image_2.JPEG]

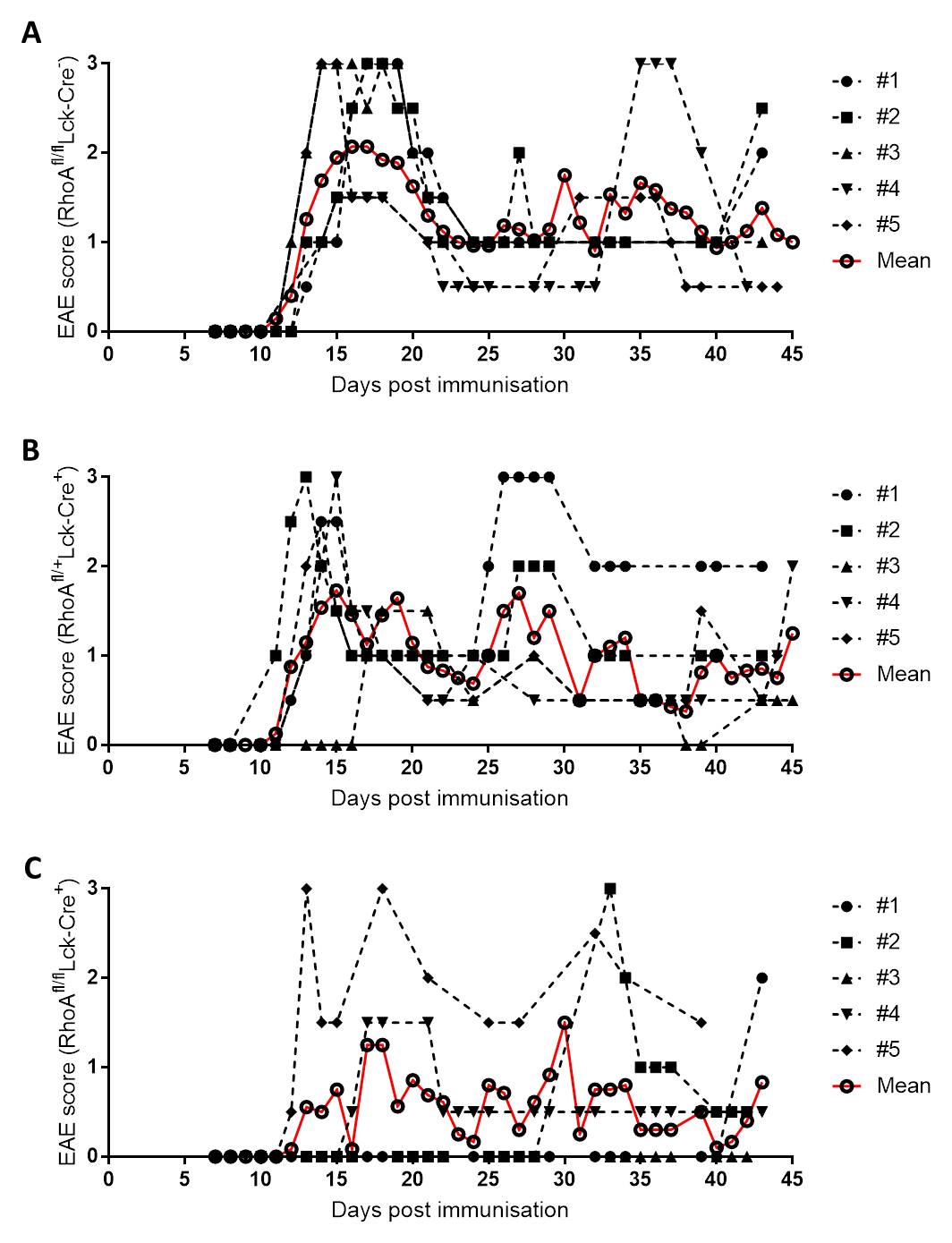

Supplement: Supplementary file 4 [file Image_3.JPEG]

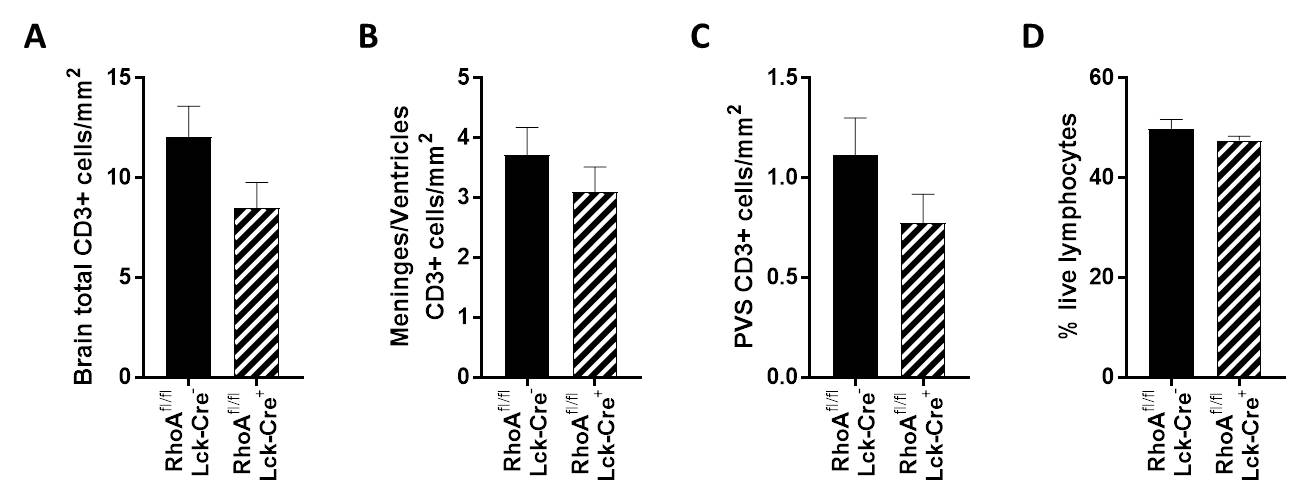

Supplement: Supplementary file 5 [file Image_4.JPEG]
